# Cell-free circulating tumor DNA in cancer

**DOI:** 10.1186/s40880-016-0092-4

**Published:** 2016-04-07

**Authors:** Zhen Qin, Vladimir A. Ljubimov, Cuiqi Zhou, Yunguang Tong, Jimin Liang

**Affiliations:** Department of Medicine, Cedars-Sinai Medical Center, David Geffen School of Medicine at UCLA, Los Angeles, California 90048 USA; School of Life Science and Technology, Xidian University, Xi’an, 710126 Shaanxi P. R. China; Keck School of Medicine, University of Southern California, Los Angeles, CA 90033 USA; Department of Pathology, Xinxiang Medical University, Xinxiang, 453003 Henan P. R. China

**Keywords:** Circulating tumor DNA, Liquid biopsy, Cancer

## Abstract

Cancer is a common cause of death worldwide. Despite significant advances in cancer treatments, the morbidity and mortality are still enormous. Tumor heterogeneity, especially intratumoral heterogeneity, is a significant reason underlying difficulties in tumor treatment and failure of a number of current therapeutic modalities, even of molecularly targeted therapies. The development of a virtually noninvasive “liquid biopsy” from the blood has been attempted to characterize tumor heterogeneity. This review focuses on cell-free circulating tumor DNA (ctDNA) in the bloodstream as a versatile biomarker. ctDNA analysis is an evolving field with many new methods being developed and optimized to be able to successfully extract and analyze ctDNA, which has vast clinical applications. ctDNA has the potential to accurately genotype the tumor and identify personalized genetic and epigenetic alterations of the entire tumor. In addition, ctDNA has the potential to accurately monitor tumor burden and treatment response, while also being able to monitor minimal residual disease, reducing the need for harmful adjuvant chemotherapy and allowing more rapid detection of relapse. There are still many challenges that need to be overcome prior to this biomarker getting wide adoption in the clinical world, including optimization, standardization, and large multicenter trials.

## Background

Cancers figure among the leading causes of morbidity and mortality worldwide, with approximately 14 million new cases and 8.2 million cancer-related deaths in 2012 [1]. The number of new cases is expected to rise by approximately 70%, from 14 million in 2012 to 22 million within the next two decades [1]. Surgery, adjuvant systemic treatment, and targeted therapies have markedly improved cancer outcomes over the past 10 years. However, many patients still die due to tumor metastasis and drug resistance [2]. Tumor heterogeneity and clonal evolution introduce significant challenges in designing effective treatment strategies [3, 4]. Different tumor cells show distinct morphologic and phenotypic features, including cellular morphology, gene expression, metabolism, motility, proliferation, and metastatic potential [5]. Heterogeneity occurs both between tumors (intertumoral heterogeneity) and within tumors (intratumoral heterogeneity). Intratumoral heterogeneity, with spatially separated heterogeneous somatic mutations and chromosomal imbalances in primary tumors or metastases, provides a tremendous challenge for cancer treatment, since examining a tissue biopsy of a portion of tumor mass could miss therapeutically relevant lesions [6]. In addition, clonal evolution in the primary tumor is partly fueled by intratumoral heterogeneity. When metastases develop, clonal evolution continues to occur under the selection pressure of anti-cancer treatments [6–8]. Molecularly targeted cancer therapies require serial monitoring of the tumor genomic makeup. However, obtaining consecutive tissue biopsies is costly, sometimes inaccessible, and associated with potential morbidity. Therefore, inability to capture spatial and temporal heterogeneity during tumor evolution results in the failure of cancer systemic treatments, requiring the development of novel approaches to detect tumor heterogeneity.

Recent progress in the analysis of blood samples for circulating tumor cells (CTC) or cell-free circulating tumor DNA (ctDNA) provides rapid, cost-effective, and noninvasive “liquid biopsy” surrogates, which give important complementary information on therapeutic targets and drug resistance mechanisms in cancer patients [9, 10]. Apoptotic or necrotic tumor cells discharge DNA fragments into the circulating blood system. These DNA fragments are called cell-free ctDNA. Cell-free DNA was initially reported by Mandel and Metais [11] in 1948 in the blood of healthy individuals. Elevated levels of ctDNA have been found in blood plasma and serum of cancer patients compared to healthy controls [12–21]. This review focuses on ctDNA and discusses the biological and technical aspects, clinical applications in cancer diagnostics, and perspectives and challenges.

## Circulating tumor cells (CTC)

CTCs are tumor cells shed into the bloodstream from primary and metastatic tumor deposits [22]. CTCs were initially detected in 1869 by an Australian physician Thomas Ashworth in a breast cancer patient’s blood [23]. Analysis of CTCs in peripheral blood of cancer patients holds great promise for the early detection of invasive cancer and the management of advanced disease. CTC detection and retrieval require elaborate methods and cumbersome processes, including highly sensitive and specific analytic methods and complex enrichment steps [24, 25]. Although techniques of CTC isolation are steadily improved with increasingly sophisticated technologies developed over the past years [26, 27], CTC identification and characterization still remain technically challenging. CTCs occur at very low concentrations of one tumor cell in the background of millions of blood cells. Particularly, patients with early-stage cancer present with extremely low CTC concentration, thus needing more sensitive assays and/or analysis of larger blood volumes, which is usually not feasible [25]. Furthermore, mechanism(s) causing CTCs to break off from the tumor are elusive; thus it is unclear whether CTCs represent the entire makeup of cancer cells in the tumor or only a subpopulation. CTCs and ctDNA likely have complementary roles as cancer biomarker since separate approaches possess distinct advantages. CTCs visualize intact cells for morphologic identification, associate with the metastatic process, and provide the unique opportunity for functional study and more comprehensive information including DNA, RNA, and protein-based molecular profiling [28]. Compared with CTCs, ctDNA is easier to isolate and more sensitive to detect. Therefore, as an alternative to CTC analysis, ctDNA appears to potentially provide superior source of genetic information, with the development of next-generation sequencing technologies.

## Origin and biological characteristics of ctDNA

Cell-free DNA is released into circulation by various pathologic and normal physiologic mechanisms. Fragments of DNA are shed into the bloodstream from dying cells during cellular turnover or apoptotic and necrotic cells [29, 30]. Under normal physiologic circumstances, apoptotic and necrotic cells are cleared by infiltrating phagocytes and cell-free DNA levels are relatively low. However, this mechanism does not act effectively in the tumor mass. Most cell-free DNA fragments are measured between 180 and 200 base pairs (bp) [30–32], suggesting apoptosis as the predominant source of cell-free DNA in the circulation [30, 33, 34]. In solid tumors, cell-free DNA can be also released through necrosis, autophagy, and other physiologic events induced by microenvironmental stress and treatment pressure [21, 35]. Unlike apoptosis, necrosis generates larger DNA fragments due to an incomplete and random digestion of genomic DNA [36]. Nevertheless, not all cell-free DNA originates from cell death. Live cells spontaneously release newly synthesized DNA as part of a homeostatically regulated system [29, 37–39]. Stimulation of lymphocytes also results in the release of large amounts of cell-free DNA in the absence of cell death [38, 40, 41]. In cancer patients, a fraction of cell-free DNA is tumor-derived and is termed ctDNA. Cancer patients generally have much higher levels of ctDNA than healthy individuals, but the levels vary widely, from 0.01% to more than 90% [12–21, 34]. The variability of ctDNA levels in cancer patients likely associates with tumor burden, stage, vascularity, cellular turnover, and response to therapy [34, 42].

Stability of cell-free DNA is not well understood. Cell-free circulating DNA appears to be rapidly cleared and the spleen, liver, and kidneys are involved in the clearance mechanism [43, 44]. The half-life of cell-free fetal DNA was previously estimated to be 16 min [45]. By using paired-end massive parallel sequencing (MPS), the same group recently studied the kinetics of cell-free fetal DNA and found that the clearance of circulating fetal DNA occurred in two phases with different kinetics [44]. The initial rapid phase had a mean half-life of approximately 1 h, whereas the subsequent slow phase had a mean half-life of approximately 13 h [44]. To date, very few studies addressed the clearance mechanism of ctDNA from plasma. Studies regarding the kinetics and clearance of circulating Epstein–Barr virus (EBV) DNA may indicate equivalent mechanisms [46–48]. Furthermore, it is also unknown whether other factors such as circadian rhythms, inflammation, or particular therapies affect ctDNA release and clearance.

Cancer harbors somatic genetic mutations and these tumor-specific alterations can be detected in ctDNA. Therefore, ctDNA carries genomic and epigenomic alterations concordant to the tumor mutational spectrum, such as point mutations, degree of integrity, rearranged genomic sequences, copy number variation (CNV), microsatellite instability (MSI), loss of heterozygosity (LOH), and DNA methylation [49]. These biological characteristics discriminate ctDNA from normal cell-free DNA and assure ctDNA as a specific biomarker that provides personalized information to detect residual disease or monitor tumor progression during therapy.

## Technologies for ctDNA analysis

Isolation of cell-free DNA for analyses of tumor-specific alterations is simple to implement from a clinical perspective. Circulating DNA is preferably extracted from plasma due to lower concentration of background wild-type DNA. The amount of cell-free DNA in serum can be 2–4 times higher than that in plasma [50], therefore, serum could be used for circulating DNA preparation. However, this is not recommended due to the possible contamination of lysed cellular DNA that would affect the relative levels of ctDNA. As described before, cell-free DNA has limited stability, thus cell-free DNA preparation should be completed promptly after blood draw.

The analysis of ctDNA is challenging and requires highly sensitive techniques due to the small fraction of tumor-specific DNA masked within background levels of wild-type cell-free DNA. Classical methods analyzing cell-free DNA include quantitative real-time polymerase chain reaction (PCR)-based, fluorescence-based, and spectrophotometric approaches [51–53]. More recently, a variety of digital genomic methods have been developed to improve identification of generic alterations in ctDNA. Digital PCR has now emerged as a sensitive tool to detect point mutations in ctDNA at low allele fractions [54], which comprises droplet-based systems [55, 56], microfluidic platforms for parallel PCR [10, 57, 58], and an approach called BEAMing (beads, emulsions, amplification and magnetics) (Table 1) [34, 59, 60].

**Table 1:** Technologies for circulating tumor DNA (ctDNA) detection

Principle of detection	Method	Type of alteration	Advantage(s)	Limitation(s)	Selected reference(s)
PCR-based	Nested real-time PCR	Known point mutations such as *KRAS*, *EGFR*, and *PIK3CA* hotspot alterations	Ease of use, lowest cost	Lower sensitivity, only detect limited genomic loci	[70]
	ARMS/Scorpion PCR				[116]
	PCR-SSCP				[117]
	Mutant allele-specific PCR				[118]
	Mass spectrometry				[119]
	Bi-PAP-A amplification				[120]
Digital PCR	BEAMing	Known point mutations, genomic rearrangements	High sensitivity	Only detect limited genomic loci	[59]
	Droplet-based digital PCR				[56]
	Microfluidic digital PCR				[10]
Targeted deep sequencing	SafeSeq	Selected SNVs, CNVs, and rearrangements across targeted regions	High sensitivity, relatively inexpensive	Less comprehensive than WES methods	[64]
	TamSeq				[57]
	Ion-AmpliSeq™				[66, 68]
	CAPP-Seq				[68]
	OnTarget				[121]
Whole-genome sequencing	Digital karyotyping	Genome-wide SNVs, CNVs, and rearrangements	Broad application	Expensive	[73]
	PARE				[70, 72, 74]

*PCR* polymerase chain reaction, *ARMS* amplified refractory mutation system, *SSCP* single-strand conformation polymorphism, *Bi-PAP-A amplification* bidirectional pyrophosphorolysis-activated polymerization allele-specific amplification, *BEAMing* beads, emulsion, amplification, and magnetics, *SafeSeq* safe sequencing system, *TamSeq* tagged amplicon deep sequencing, *CAPP-Seq* cancer personalized profiling by deep sequencing, *PARE* personalized analysis of rearranged ends, *KRAS* Kirsten rat sarcoma viral oncogene homolog, *EGFR* epidermal growth factor receptor, *PIK3CA* phosphatidylinositol-4,5-biphosphate 3-kinase, catalytic subunit alpha, *SNV* single-nucleotide variants, *CNVs* copy number variations, *WES* whole-exome sequencing

Next-generation sequencing technologies are currently being applied to plasma DNA analysis. These high-throughput, low-cost, sequencing technologies identify widespread ctDNA alterations across wide genomic regions [61–63]. Targeted deep sequencing approaches have been used to analyze specified genomic regions in plasma DNA, including PCR-based targeted deep sequencing such as TamSeq [10, 57], SafeSeq [64, 65], and Ion-AmpliSeq™ [66, 67] and capture-based targeted deep sequencing such as CAPP-Seq [68] (Table 1). Remarkably, whole genome sequencing provides novel opportunities for comprehensive characterization of the alteration profiles, not just limited to predefined or existing mutations in plasma DNA [69]. Genome-wide detection of chromosomal rearrangements and CNVs can be characterized in ctDNA, serving as tumor biomarkers with excellent sensitivity and specificity [70, 71]. Two genome-wide methods, personalized analysis of rearranged ends (PARE) [70, 72] and digital karyotyping [73] can be applied to ctDNA detection (Table 1). PARE is a method for identifying specific somatic rearrangements in tumor tissue and subsequently developing PCR-based assays to detect these tumor biomarkers in the circulation [72, 74]. Digital karyotyping is a genome-wide technique used to quantify the DNA copy number and novel sequences on a genomic scale. It has been applied to detect previously uncharacterized chromosomal changes and exogenous sequences in human tumors [73, 75, 76]. Moreover, recent implementation of whole-genome sequencing allows direct application to ctDNA analysis, and has provided an unprecedented view of somatic chromosomal alterations and CNVs on a genome-wide scale [74, 77, 78]. Undoubtedly, with continuous improvements in the sensitivity of genomic approaches, next generation sequencing techniques will play a pivotal role in ctDNA analysis for future clinical applications.

Evaluation of cell-free DNA integrity index is a different approach to identify ctDNA alterations and constitutes an independent indicator different from any specific genomic changes. DNA integrity index is measured as the ratio of long to short DNA fragments. Circulating cell-free DNA released from apoptotic cells is uniformly truncated into 185- to 200-bp fragments [79, 80], whereas cell-free DNA released from necrotic tumor cells varies in length, which may lead to elevation of DNA with long fragments in plasma [30] or serum [81]. A study by Leon et al. [82] suggested that the cell-free DNA concentration was significantly increased in cancer patients compared with that in healthy individuals. Similar findings have also been demonstrated in several cancers such as periampullary cancer [80], breast cancer [81], colorectal cancer [80], esophageal cancer [83], head and neck cancer [84], renal cancer [85], melanoma [86], and prostate cancer [87].

## Clinical applications of ctDNA

### Tumor genotyping: detection of genetic and epigenetic alterations

In principle, ctDNA fragments contain genetic defects identical to those of tumor tissues, including point mutations, rearrangements, amplifications, MSI, LOH, and tumor-associated DNA methylation [88]. To perform blood-based tumor genotyping assays by using ctDNA will be greatly beneficial for guiding personalized cancer treatment (Table 2).

**Table 2:** Potential application of ctDNA in clinical oncology

Cancer screening	Localized cancer	Metastatic cancer	Refractory cancer
Early diagnosis and early intervention	Identifying specific genomic alterations to guide therapeutic selection, monitoring tumor burden and therapeutic responses, detecting minimal residual disease, assessing risks of dissemination and recurrence	Early identification of relapse and treatment resistance, guidance of treatment selection, monitoring therapeutic responses	Understanding mechanism of resistance, determining new treatment

#### Detection of tumor-specific mutations and CNVs

Two separate studies in 1994 first described Kirsten rat sarcoma viral oncogene homolog (KRAS) and neuroblastoma RAS viral (v-ras) oncogene homolog (NRAS) mutations in the blood of pancreatic carcinoma [89] and leukemia patients [90]. During the past two decades, abundant mutations have been detected in the ctDNA of patients with various types of cancer [49]. Next generation sequencing has been directly applied to ctDNA analysis. Dawson et al. [10] used targeted or whole-genome sequencing to assess genetic mutations in tumors samples from 30 metastatic breast cancer patients, and designed personalized assays to quantify ctDNA genetic alterations. They found that ctDNA levels showed a greater dynamic range and associated with changes in tumor burden. Leary et al. [74] published the first whole-genome sequencing analysis of ctDNA. They successfully identified ctDNA in concentrations of less than 1% with a sensitivity >90% and a specificity >99%. Single-nucleotide variants (SNVs) and CNV were detected in all advanced-stage cancer patients, but not in healthy subjects [74]. Recently, Chan et al. [77] applied shotgun MPS of plasma DNA from cancer patients to scan the cancer genome and achieved the genome-wide profiling of CNVs and point mutations. Concordant genome-wide SNVs have been identified between tumor tissues and pre-surgical cell-free DNA. Most importantly, the structural alterations in plasma DNA entirely disappeared after surgery. Moreover, the CNV profile detected in ctDNA was derived from three primary tumor mixtures in a cancer patient, indicating that ctDNA sequencing is a valuable approach for studying tumor heterogeneity [77].

#### Detection of MSI and LOH in ctDNA

MSI, such as LOH, is frequently found in tumor tissues. Detection of MSI and LOH in ctDNA was first reported by Nawroz et al. [84] in 1996. To date, similar studies have been completed in breast, brain, colorectal, ovarian, and prostate cancers [49]. Recently, a study involving a large cohort of breast cancer patients (*n* = 388) showed that high LOH frequencies were associated with the aggressiveness of breast cancer, and in particular, the observed *CCND2* loss was a strong indicator of an unfavorable prognosis [91].

#### Detection of tumor-associated DNA methylation in ctDNA

DNA methylation plays pivotal roles in gene regulation and genome stability. Genes with high levels of 5-methylcytosine in the promoter region are transcriptionally silent. This process is often dysregulated in tumor cells. Aberrations of DNA methylation in the gene promoter region or in the non-coding genomic sequences are associated with tumor initiation, dissemination and metastasis establishment, and progression [92]. The status of DNA methylation is very stable, even in the circulation; thus it can be assessed to monitor tumor-related processes. Aberrant DNA methylation has been first detected in the plasma and serum of lung [93], breast [94], and liver cancer patients in 1999 [95]. Since then, extensive studies have indicated the potential of ctDNA methylation as a diagnostic and prognostic marker for cancer patients [49, 96–98]. Recently, a study identified genome-wide ctDNA methylation in esophageal cancer patients, and observed highly concordant methylation profiles between ctDNA and corresponding tumor tissues [99]. Differential ctDNA methylation profiles were characterized to distinguish esophageal adenocarcinoma, precursors, and controls [99]. The study suggested that ctDNA can produce excellent methylation profiling on a genome-wide scale and serve as a useful tool to develop methylation-based biomarkers for clinical application.

### Monitoring tumor burden and therapeutic responses

Dynamics of ctDNA has been investigated in various solid malignancies for the relationship between ctDNA levels, tumor burden, and therapeutic responses [10, 34, 70, 72, 100, 101]. Protein biomarkers are conventionally used in cancer diagnosis and in the assessment of therapeutic responses, such as carcinoembryonic antigen (CEA), prostate-specific antigen (PSA), cancer antigen (CA) 19-9, and CA-125. Unfortunately, the specificity and reliability of these protein biomarkers are not satisfactory, and many malignancies even do not have any reliable protein biomarker [102, 103]. ctDNA carries comprehensive, inherently specific, and highly sensitive information, and thus possesses distinctive advantage over conventional protein biomarkers. Studies in melanoma [104, 105], breast [10], ovarian [57], and colon cancers [34, 106] have solidified the potential applications of ctDNA to monitor tumor burden dynamically and precisely during treatment process. ctDNA levels increased rapidly with disease progression and declined correspondingly after successful treatment [10, 34, 57, 104, 105]. Quantitative assessment of ctDNA levels could also be an important indicator of prognosis (Table 2). Some preliminary data supported an association between ctDNA levels and prognosis in cancer patients with advanced-stage disease [10, 107–109].

Treatment resistance is a major problem in the care of cancer patients. ctDNA can effectively assess the emergence of mutations associated with treatment resistance [110–114]. The molecular alterations of *KRAS* are causally associated with the onset of acquired resistance to anti-EGFR treatment of colorectal cancers. Detection of *KRAS* variants in ctDNA of patients receiving anti-EGFR therapies can identify relapse 10 months before radiographic documentation of disease progression [111]. Furthermore, by using whole-exome sequencing, serial ctDNA analysis can provide an unbiased and comprehensive assessment of genomic alterations during the acquisition of treatment resistance [69].

ctDNA analysis can ultimately provide a global picture of genetic alterations, including the dynamic changes of the mutation profile as well as tumor heterogeneity and clonal evolution throughout the course of cancer treatment (Table 2). This global picture can help for designing combination treatments to minimize therapeutic resistance.

### Monitoring minimal residual disease

ctDNA can be potentially applied to detect minimal residual disease after surgery or therapy with curative intent [100, 115]. In certain type of cancers, respective surgery alone cures a large portion of patients with early-stage, localized tumor. However, no effective approach can discriminate which patients are cured and which have residual disease that will result in recurrence. Therefore, some patients potentially cured by surgery still receive adjuvant chemotherapy unnecessarily since lack of information. ctDNA is a potential marker for residual disease after resection to identify individuals at risk of relapse (Table 2). Studies show that assessment of tumor-specific mutations in plasma DNA following surgical resection can identify individuals with residual disease [89], and detect disease recurrence [84, 85]. The early prediction of recurrence will allow effective treatment strategies to be introduced at a time when disease burden is still minimal.

## Challenges of ctDNA analysis on the path to clinical utility

As a noninvasive “liquid biopsy,” ctDNA is a promising biomarker that provides highly specific and complementary information in the diagnosis, prognosis, and management of cancer treatment. With the rapid advances and the decrease in the cost of next-generation sequencing, the technology brings a new alternative for unbiased ctDNA exploration. However, clinical routine practice is slow to adopt this approach since several challenges remain despite the remarkable progress in recent years. Most ctDNA studies focused on advanced-stage cancers with relatively high concentrations of ctDNA. Detailed experiences with early-stage cancer and low concentrations of ctDNA are lacking. High levels of normal DNA aggravated during inflammation and injury could dilute ctDNA and interfere ctDNA detection. Therefore, ctDNA analysis in some clinical settings may result from detection of nonprogressing benign lesions. In addition, the diverse technologies of ctDNA analysis need to be optimized and different platforms need to be standardized, and the appropriate analytic and clinical validity needs to be demonstrated, to control the pre-analytic phase and obtain robust and reproducible results. Most importantly, critical clinical standards need to be established, and well-designed and sufficiently powered multicenter clinical trials involving large cohorts of patients and controls are required to validate ctDNA as clinical biomarker.
